# Effects of subthalamic nucleus deep brain stimulation on the speech of Spanish-speaking Parkinson’s disease patients during the first year of treatment

**DOI:** 10.1590/2317-1782/20242023194en

**Published:** 2024-09-02

**Authors:** Nicolás Castillo-Triana, Maryluz Camargo-Mendoza, Óscar Bernal-Pacheco

**Affiliations:** 1 Departamento de Comunicación Humana, Facultad de Medicina, Universidad Nacional de Colombia – UNAL - Bogotá, Colombia.; 2 Unidad de Neurocirugía Funcional, Instituto Roosevelt - Bogotá, Colombia.

**Keywords:** Parkinson’s Disease, Deep Brain Stimulation, Dysarthria, Speech, Voice, Doença de Parkinson, Estimulação Cerebral Profunda, Disartria, Fala, Voz

## Abstract

**Purpose:**

To describe the effects of subthalamic nucleus deep brain stimulation (STN-DBS) on the speech of Spanish-speaking Parkinson's disease (PD) patients during the first year of treatment.

**Methods:**

The speech measures (SMs): maximum phonation time, acoustic voice measures, speech rate, speech intelligibility measures, and oral diadochokinesis rates of nine Colombian idiopathic PD patients (four females and five males; age = 63 ± 7 years; years of PD = 10 ± 7 years; UPDRS-III = 57 ± 6; H&Y = 2 ± 0.3) were studied in OFF and ON medication states before and every three months during the first year after STN-DBS surgery. Praat software and healthy native listeners’ ratings were used for speech analysis. Statistical analysis tried to find significant differences in the SMs during follow-up (Friedman test) and between medication states (Wilcoxon paired test). Also, a pre-surgery variation interval (PSVI) of reference for every participant and SM was calculated to make an individual analysis of post-surgery variation.

**Results:**

Non-significative post-surgery or medication state-related differences in the SMs were found. Nevertheless, individually, based on PSVIs, the SMs exhibited: no variation, inconsistent or consistent variation during post-surgery follow-up in different combinations, depending on the medication state.

**Conclusion:**

As a group, participants did not have a shared post-surgery pattern of change in any SM. Instead, based on PSVIs, the SMs varied differently in every participant, which suggests that in Spanish-speaking PD patients, the effects of STN-DBS on speech during the first year of treatment could be highly variable.

## RESUMO

**Objetivo:**

Descrever os efeitos da estimulação cerebral profunda do núcleo subtalâmico (ECP-NST) na fala de pacientes com doença de Parkinson (DP) falantes de espanhol durante o primeiro ano de tratamento.

**Método:**

As medidas de fala (MFs): tempo máximo de fonação, medidas acústicas de voz, velocidade de fala, medidas de inteligibilidade de fala e taxas de diadococinesia oral de nove pacientes colombianos com DP idiopática (quatro mulheres e cinco homens; idade = 63 ± 7 anos; anos de DP = 10 ± 7 anos; UPDRS-III = 57 ± 6; H&Y = 2 ± 0,3) foram estudados nos estados de medicação OFF e ON antes e durante um ano após a cirurgia de ECP-NST a cada três meses. O software Praat e as avaliações de ouvintes nativos saudáveis foram utilizados para análise de fala. A análise estatística tentou encontrar diferenças significativas entre as avaliações pré-operatórias e de seguimento (Teste de Friedman) e entre os estados de medicação (Teste pareado de Wilcoxon OFF/ON). Também, um intervalo de variação pré-cirúrgico (IVPC) para cada participante e MF foi calculado para fazer uma análise individual da variação durante o seguimento.

**Resultados:**

Não foram encontradas diferenças significativas ao longo do seguimento ou entre os estados medicamentosos em as MFs. No entanto, individualmente, com base nos IVPCs, as MFs exibiram: nenhuma variação, variação inconsistente ou variação consistente durante o seguimento em diferentes combinações, dependendo do estado da medicação.

**Conclusão:**

Os participantes não tiveram um padrão compartilhado de variação de fala durante seguimento. As MFs exibiram diferentes padrões de variação em cada participante, que sugerem que em pacientes com DP de língua espanhola, os efeitos da ECP-NST durante o primeiro ano de tratamento na fala podem ser altamente variáveis.

## INTRODUCTION

Parkinson's Disease (PD) is a neurodegenerative disorder characterized by resting tremor, bradykinesia, akinesia, postural instability, freezing, and rigidity^(1-3)^. PD patients also can experience cognitive^(4,5)^, psychiatric^(6)^, swallowing^(7-9)^, and speech impairment^(9,10)^. Speech signs in PD are related mainly to respiratory, phonatory, articulatory, and prosodic dysfunction. Patients can experience hypophonia and short phrases related to alterations of breath support for speech^(11-13)^. Also, they can exhibit voice problems like breathy voice, roughness, hoarseness, vocal tremor, and vocal pitch alterations^(13,14)^. At the articulation level, articulatory imprecisions are well documented^(15)^. Monotonous and naturalness-reduced speech is the main prosodic alteration among this population^(16,17)^.

Although PD management is commonly pharmacological^(18)^, some patients who experience highly disabling motor symptoms, which are not completely controlled with levodopa, or who experience levodopa side effects benefit from deep brain stimulation (DBS)^(18-20)^. DBS aims to ameliorate or counteract pathological neural activity by delivering a localized continuous electrical current into specific target brain regions^(21,22)^ such as internal globus pallidus^(23)^, zona incerta^(24)^, or the subthalamic nucleus^(23,25)^.

The effects of Subthalamic Nucleus DBS (STN-DBS) on the speech of PD patients are heterogeneous^(26,27)^, partly because of the variety of study designs. Currently, evidence about the speech outcome in STN-DBS-treated PD patients comes from (a) studies that compare the speech of pharmacologically treated patients versus STN-DBS-treated ones^(28-33)^, (b) studies that compare speech before and after STN-DBS implantation surgery^(34-41)^, (c) studies that compare speech after surgery in ON and OFF STN-DBS states^(42-51)^, (d) and from some studies that explore patients' self-perceptions of speech changes after STN-DBS onset^(52-55)^.

Beneficial and adverse effects of STN-DBS on acoustic and perceptual speech parameters have been identified when STN-BDS-treated patients are compared with pharmacologically treated ones. For example, scores in GRBAS voice scale parameters^(28)^ and in dimensions of dysarthric speech, such as short rushes of speech, hypernasality, and consonant distortions, were worse in STN-DBS-treated patients^(29)^. Similarly, Tanaka et al.^(30)^ observed that these patients had worse (higher) values of Jitter, Shimmer, noise-to-harmonics ratio (NHR), vocal tremor, and degree of voicelessness. In contrast, some authors^(31,32)^, using a non-linear method of voice analysis, found that STN-DBS-treated patients had better voice quality than pharmacological-treated patients. Equally, better performance on Vowel Space Area (VSA), a measure indicative of articulation ability, is reported in STN-DBS-treated patients^(33)^.

On the other hand, pre-/post-surgery speech comparisons have revealed that after STN-DBS device implantation, voice intensity^(34,35)^, pitch variation, pitch range^(36)^, oral diadochokinesis rate^(37),^ and Long-Term Average Spectrum (LTAS) measures of voice^(34)^ are better. Nevertheless, similar studies have found that speech intelligibility, or how well a listener can accurately recover an acoustic signal from a speaker^(38)^, and vocal quality diminish after STN-DBS surgery^(39-41)^.

Similar findings are observed when the ON STN-DBS state is compared with the OFF STN-DBS state. In the ON stimulation versus OFF stimulation state, PD patients show higher voice intensity^(42)^, better acoustics voice parameters -lower values of Jitter, Shimmer, NHR, and vocal tremor-^(43-45)^, higher maximum phonation time (MPT)^(45,46),^ higher speech rate, higher oral diadochokinesis rate^(45)^, larger VSA^(47)^ and worse speech intelligibility^(41)^. Other studies have not found speech variation related to STN-DBS^(48-51)^, and studies about patients' self-perception of voice and speech changes after STN-DBS surgery show adverse effects in most cases^(52-55)^.

The beneficial effects of STN-DBS on speech are attributed to oral bradykinesia and hypokinesia reduction caused by electrical stimulation^(33)^. Studies show better performance of speech mechanism structures in patients treated with STN-DBS during speech^(56,57)^ and nonspeech tasks^(58)^. Likewise, the adverse effects of STN-DBS on speech are probably the result of electrical current diffusion toward non-intended brain regions^(59-61)^. Spastic dysarthria and strained voice quality emerge only when STN-BDS is ON (not in OFF state) and are not observed in pharmacologically treated patients^(40)^.

Above mentioned studies have investigated speakers of Chinese^(51)^, English^(34)^, French^(45,47)^, Italian^(43)^, Japanese^(33,40,41)^, Portuguese^(49)^, Swedish^(37,39,42)^, and Turkish^(48)^, so Spanish-speaking PD patients are underrepresented. Also, conclusive results cannot be extracted from available evidence and applied to speakers of other languages due to methodological disparities among studies^(26)^. Consequently, linguistically appropriate data about STN-DBS effects on speech becomes relevant to Spanish-speaking PD patients. Finally, many studies have been concerned with documenting the long-term effects (after one year or more) of STN-BDS on speech^(62-64)^ and have studied only specific speech parameters^(37,42-50)^, so it is also needed to understand whether reported long-term changes in isolated speech measures can be detected early after STN-DBS implantation.

This study aimed to describe the effects of STN-DBS on the speech of Spanish-speaking PD patients during the first year of treatment using a comprehensive set of speech measures.

## METHODS

### Participants

The Instituto Roosevelt Ethics Committee Research approved this study (Letter N° 2020021204-002). Participants received the STN-DBS implantation surgery at this hospital between February and September 2020. Participants had no history of other medical conditions, different than PD, that could impair speech. No participant had evident hearing lossand just one participant (p09) had the diagnosis of mild cognitive impairment. None of the participants attended speech therapy at the study enrolment. Pre-surgery speech impairment was not an inclusion or exclusion criterion.

Initially, thirteen native Colombian Spanish-speaking patients with idiopathic PD accepted their participation through written informed consent. Two participants retired from the study because of health deterioration, another because of STN-DBS device organic incompatibility, and another due to health insurance issues that prevented surgery. Four women and five men were the definitive participants (Table 1). The Unified Parkinson's Disease Rating Scale-part III (UPDRS-III) and Hoehn and Yahr Scale (H&Y) were rated by a neurologist movement disorders specialist. UPDRS-III ratings were made in the OFF-medication state on the same day of the surgery prior to the procedure. Levodopa equivalent dose (LED) was calculated according to Tomlinson et al.^(65)^.

**Table 1:** Pre-surgery study participant's characterization

Participant	Sex	Age (years)	Years of PD	LED (mg)	UPDRS-III	H&Y
p01	F	57	9	488	51	2
p02	F	60	11	648	57	2
p03	M	69	8	798	56	2
p04	F	66	6	728	55	3
p05	M	67	17	945	56	2
p06	M	68	8	798	52	2
p07	M	49	9	975	72	2
p08	F	70	11	940	53	2
p09	M	61	8	735	61	2
Mean (SD)	-	63(7)	10(7)	784(158)	57(6)	2(.3)

Caption: F = female; M = male; LED = Levodopa equivalent dose; UPDRS-III = Unified Parkinson's Disease Rating Scale-part III; H&Y = Hoehn and Yahr Scale; SD = standard deviation

### STN-DBS device implantation

Six participants received the STN-DBS device *Activa RC model 37612* (Medtronic), and the others (p01, p03, and p07) the STN-DBS device *DB- 5552-1A Vercise DBS* (Boston Scientific). The implantation was bilateral in all the cases. Surgery technique is detailed elsewhere^(66,67)^.

### Follow-up

Speech assessments were made twice before STN-DBS surgery (at 72 ± 55 days and 9 ± 6 days before) and at three (100 ± 10 days), six (189 ± 10 days), nine (280 ± 6 days), and twelve months (382 ± 27 days) after surgery in OFF (OFF-med) and ON (ON-med) medication states. Also, after surgery, STN-DBS was always in ON. The OFF-med was considered the period before the first levodopa dose consumption in the morning (patients were under nocturn levodopa withdrawal), and the ON-med was considered one hour after this levodopa dose consumption^(68,69)^. For speech assessments, patients also withdrew long-action PD medication, and they returned to their regular medication immediately after ON-med assessments.

#### Recording equipment

Participants were audio recorded with a ZOOM H4n digital audio recorder -sample rate 44.1 KHz- (Zoom Corp.) and a Shure SM35 headset professional microphone (Shure Inc.) placed five centimeters from the mouth corner. The resulting audio files were saved in wav format.

#### Speech assessment protocol

Speech assessment sessions were made in a quiet room of participants' homes. These sessions were early in the morning to alter the less possible habitual participants' medication schedules. Participants performed the following speech tasks during pre- and post-surgery assessments:

1. Sustained phonation of /a/ (3 times). Participants were asked to “take a breath and then say 'ahh' as long as possible in your normal voice.”
2. Reading aloud a Spanish phonetically balanced text^(70)^ (Supplementary Material – Section 1)
3. Monologue task. Participants were asked to “tell me everything you did yesterday, from morning to night, as detailed as possible.”
4. Oral diadochokinesis with /pa/, /ta/, /ka/, and /pata'ka/ (3 times per stimulus). Participants were asked to “repeat after me, as fast as possible, this (intended stimulus)”

#### STN-DBS settings

Participants underwent periodic STN-DBS settings adjustments by their neurologist to improve motor performance as part of every participant's independent study treatment plan. This data was extracted from patients' medical records (Supplementary Material - Table 1S).

### Speech measures

Patients' audio recordings, where speech measures (SMs) were extracted, were analyzed only when one year of follow-up was completed for all participants. Nineteen SMs were calculated blindly by a speech-language pathologist with experience in dysarthric speech analysis. The assessment session, participant, and medication information of every analyzed audio recording was revealed when all SMs were calculated for all participants. The SMs were grouped into five categories: acoustic voice measures, Maximum Phonation Time (MPT), speech intelligibility (SI) measures, speech rate (SR), and oral diadochokinesis rates.

#### Acoustic voice measures

Fundamental frequency (*f*o), standard deviation of *f*o (SD*f*o), vocal intensity, Jitter (local), Jitter (RAP), Shimmer, noise-to-harmonics ratio (NHR), harmonics-to-noise ratio (HNR), and smoothed cepstral peak of prominence (CPPS) were calculated from the three central seconds of every single sustained phonation. Long-Term Average Spectrum (LTAS) measures were extracted from three segments of the reading-aloud task (Supplementary Material – Section 1). Analysis was made using Praat^(71)^. CPPS and LTAS measures (slope and tilt-trendline [t-t] methods) were calculated using an established procedure^(72)^. The other acoustic voice measures were established through Praat's *voice report* option. All acoustic voice measures were registered as the average from the three sustained phonation trials per participant or the three reading-aloud task segments (LTAS measures) from every assessment session and medication state.

#### MPT

MPT was calculated as the average duration of the three sustained phonation trials. The start and the end of the productions were Praat's oscillogram-based. MPT was calculated from phonation start until the participant's first phonatory interruption.

#### SI measures

Two SI measures were studied: the percentage of correctly identified words (CIW) and the grade of speech intelligibility (GI). GI was rated according to a Likert scale of nine points, 1 = '*not understandable at all'* and 9 = *'completely understandable'* as in Moya-Galé et al.^(73)^, and CIW was calculated using the next equation: CIW = (words transcribed adequately per sentence / total words per sentence) x 100%^(74,75)^

SI measures were determined from sentences extracted from monologue task audio recordings (sentences average long 9 ± 6 words). Three sentences were extracted per participant from every assessment session and medication state (n = 324). These sentences were transformed into independent audio files with Praat and were intensity normalized with Audacity (Muse Group). The SI rating process was made in a speech laboratory by six native Colombian Spanish speakers, Speech-Language Pathology students of the first year, unfamiliar with dysarthric speech (listener's average age 21 ± 3 years). Listeners accepted their participation by informed consent signing. They passed a hearing screening test made with the Android mobile application Hearing Test version 2.0.26^(76)^ using a Samsung Galaxy A201 Smartphone (Samsung Electronics) and Essens™ headphones (BeDigital S.A.) under noise-controlled conditions (noise level < 45 dB SPL), according to an Extech digital sonometer model 407730 (Extech Instruments).

Every two listeners listened to the sentences of three different participants (n = 108). Additionally, they relistened ten percent of these sentences (n= 11), randomly selected, to assess intra- and inter-listener reliability of ratings. During the SI rating process, audio files were presented randomly from an HP Pavilion laptop (HP Inc.) connected to an Altec Lansing Multimedia Computer Speaker System ACS33 (Altec Lansing Technologies Inc.). Listeners placed one meter of distance in front of the speakers.

Listeners were asked to write down everything they listened to (word and nonword) to calculate the CIW percentage. Also, immediately after that, they had to assign a GI to every transcribed sentence. They were indicated to assign GI 1 if they cannot understand any word, GI 9 in case they can understand every sentence word easily, and the other scale numbers taking GI 1 and GI 9 as reference. Before starting the rating process, listeners wrote down a Spanish pangram to collect a writing sample in case of doubt during sentence transcriptions reading. Also, two trial sentences (sentences different from the study) were listened to and transcribed, and a GI was assigned to exemplify the procedure. Every sentence was reproduced once and on-demand, so every sentence was not reproduced more than twice.

#### SR

Speech rate (SR) was calculated using the same sentences to extract SI measures. These sentences were orthographically transcribed and, after that, processed with a Spanish syllable counter tool^(77)^. SR scores were calculated using the next equation: SR = (syllables per sentence/seconds spent per sentence). Seconds spent per sentence were determined with Praat, from the beginning to the end of every sentence Praat oscillogram selection.

#### Oral diadochokinesis rates

Alternate (AMR) and sequential motion rate (SMR) were calculated according to the next equations: AMR = (# of /pa/, /ta/, or /ka/ repetitions /seconds spent performing repetitions); SMR = (# of syllables in /pata'ka/ repetitions/ seconds spent performing repetitions). The number of syllables repeated, and seconds spent performing repetitions were determined through Praat analysis. The average result of three trials per participant in every assessment session and medication status was considered.

### Dysarthria severity

Dysarthria severity assessment was blind (the assessment session and the participant were unknown to the evaluator), was made at the beginning and the end of follow-up in OFF-med by a speech-language pathologist with experience in dysarthric speech analysis, using a translated version of the Dysarthria Rating Scale (DRS) proposed by Duffy (Table 2S, Supplementary Material)^(78)^, since currently there are no validated Spanish language dysarthria assessment tools. The DRS assigns a grade of severity (0 = normal; 1 = mild; 2 = moderate; 3 = marked; 4 = severe) to 47 dysarthric speech dimensions grouped into eight categories. The DRS has a total score (DRS-TS) that results from the sum of the subtotal scores per category, the higher the DRS-TS, the higher the dysarthria severity. This study used a DRS-adapted version of seven categories and 41 dimensions. The excluded category was 'Other', composed of six dimensions, of which three were related to oral diadochokinesis analysis (oral diadochokinesis was assessed objectively in this study), and the other three dimensions were related to infrequent PD patients' speech signs (simple vocal tics, palilalia, and coprolalia).

**Table 2:** Participants' SMs in OFF-med during follow-up

Speech measure	pre-surgery		3 mps		6 mps		9 mps		12 mps	
	Median	IQR (Q1-Q3)	Median	IQR (Q1 – Q3)	Median	IQR (Q1 – Q3)	Median	IQR (Q1 - Q3)	Median	IQR (Q1 – Q3)
MPT (s)	11.6	4.1 (8.7 – 12.8)	11	10 (7.3 – 17.4)	14.2	10.6 (7.4 – 18)	9.6	7.3 (6.7 – 14)	11.5	4.3 (9.5 – 13.8)
*fo* (Hz)	142.9	77.8 (111 – 188.8)	137.7	40.1 (128.5 – 168.6)	136.2	45.4 (131.4 – 176.8)	137.9	76.8 (134 – 210.8)	153.4	45.5 (140.2 – 185.7)
SD*f*o	6.5	16.3 (2.8 – 19)	3.4	15 (1.8 – 16.8)	4.6	29.6 (2.5 – 32.1)	6.5	6.7 (1.8 – 8.5)	2.8	4.7 (2.2 – 6.9)
Intensity (dB)	58.5	8.4 (54.9 – 63.3)	59.2	10.1 (52 – 62.1)	61.6	3.4 (58.8 – 62.2)	60.9	4.4 (59.5 – 63.9)	65.4	3 (64.4 – 67.4)
Jitter: local (%)	.51	.21 (.39 – .60)	.55	.57 (.26 – .83)	.46	.13 (.37 – .50)	.51	.44 (.44 – .88)	.40	.25 (.27 – .52)
Jitter: RAP (%)	.25	.11 (.21 – .32)	.30	.34 (.13 – .47)	.21	.12 (.17 – .29)	.31	.25 (.22 – .47)	.15	.16 (.13 – .29)
Shimmer: local (%)	3.5	1.2 (2.7 – 3.9)	2.9	1.5 (2.3 – 3.8)	2.7	2.1 (2.2 – 4.3)	3.5	2.9 (2.3 – 5.2)	2.6	1.3 (1.5 – 2.8)
NHR	.014	.018 (.007 – .025)	.023	.044 (.009 – .053)	.024	.010 (.017 – .027)	.020	.052 (.015 – .067)	.014	.010 (.007 – .017)
HNR (dB)	20	4.3 (19 – 23.3)	18.1	5.3 (16.5 – 21.8)	17.6	1.9 (17.1 – 18.9)	19	5.9 (15.9 – 21.8)	20.4	3.9 (18.7 – 22.5)
CPPS (dB)	14.9	3 (13.8 – 16.8)	14.6	6.1 (12.3 – 18.5)	16.4	2.7 (15.4 – 18.1)	15	3.4 (14.8 – 18.2)	17.1	5.6 (15.3 – 21)
LTAS-slope (dB)	-27.7	4.4 ([-30.6] – [-26.2])	-28.1	6.7 ([-31.8] – [-25.1])	-28.1	2.7 ([-28.9] – [-26.2])	-29.5	3.3 ([-30.2] – [-26.9])	-27.7	5.2 ([-31.3] – [-26.1])
LTAS-t-t (dB)	-19.6	13.5 ([-24.8] – [-11.3])	-14.1	18.9 ([-27.8] – [-8.9])	-14.2	12.8 ([-18.7] – [-5.9])	-24.4	12.2 ([-28.4] – [-16.1])	-22.4	10.3 ([-29.1] – [-18.8])
CIW (%)	90	20.7 (76.5 – 97.2)	87	42.7 (54.3 - 97)	84.2	30.9 (62.3 – 93.2)	62.1	26 (53.9 – 79.9)	67.5	16.5 (57.9 – 74.4)
GI	8	2 (7 – 9)	7	2 (5 – 7)	7	1 (6 – 7)	6	4 (4 – 8)	6	4 (4 – 8)
SR (sil/s)	5.9	1.4 (5 – 6.4)	6.2	1.4 (5.5 – 6.9)	6	0.7 (5.6 – 6.3)	5.8	1.9 (4.6 – 6.5)	6.1	1.1 (5.4 – 6.5)
/pa/ (sil/s)	6.4	0.7 (5.9 – 6.6)	6	0.5 (5.7 – 6.2)	6.1	1 (5.4 – 6.4)	5.6	0.5 (5.4 – 5.9)	5.5	1.2 (5.1 – 6.3)
/ta/ (sil/s)	6.4	1.2 (5.5 – 6.7)	5.5	1 (5.1 – 6.1)	5.3	1.4 (5 – 6.4)	5.3	1 (4.9 – 5.9)	5.2	1.7 (4.5 – 6.2)
/ka/ (sil/s)	5.8	0.8 (5.5 – 6.3)	5.5	0.6 (5.3 – 5.9)	5.2	0.7 (4.8 – 5.5)	5	0.5 (4.7 – 5.2)	4.8	1.2 (4.5 – 5.7)
/pata´ka/ (sil/s)	6.4	0.4 (6.3 – 6.7)	6.7	1.5 (5.9 – 7.4)	6.4	0.9 (5.8 – 6.7)	6.4	1.1 (6 – 7.1)	6.2	1.3 (5.6 – 6.9)

Caption: IQR = interquartile range; Q1 = quartile 1; Q3 = quartile 3: mps = months post-surgery; sil/s = syllables/ second

### Data analysis

Statistical analysis was made with R^(79)^. The median and interquartile range (IQR) of speech measures were determined (first and second pre-surgery assessment sessions data were analyzed together). Friedman's test was made to establish any speech change between assessment sessions at the group level. When applied, *post hoc* analyses were made through multiple comparisons applying Bonferroni correction. Likewise, differences between medication states (ON/OFF) were tested through a Wilcoxon-paired test.

A pre-surgery variation interval (PSVI) of reference for every participant and SM was calculated to make an individual analysis of variation during post-surgery follow-up. These PSVIs were calculated based on the mean value of every SM per participant at pre-surgery assessments (assessments 1 and 2) ± 1.5 standard deviations like in other studies^(72,80)^. Finally, intra- and inter-listener reliability of the percentage of CIW estimations were tested through the intraclass correlation coefficient method (ICC) and GI ratings through Cohen's kappa coefficient method (CKC).

## RESULTS

### Dysarthria severity

According to DRS-TS, before STN-DBS surgery, participants had three dysarthria levels: low (DRS-TS = 1), middle (DRS-TS = 4), and high (DRS-TS = [6 – 8]). Participants with high DRS-TS had breathing and articulation impairment and more severe impairment in prosody, voice quality, pitch, and loudness than others. Participants with middle DRS-TS had impairment in pitch, loudness, voice quality, and prosody without articulation or breathing impairment. Finally, the participant with low DRS-TS just had voice quality impairment. After one year of STN-DBS, the DRS-TSs exhibited little change in most participants, except for one participant (p09) who showed exacerbation of articulation difficulties after surgery (Table 3S, Supplementary Material).

**Table 3:** Participants' SMs in ON-med during follow-up

Speech measure	PRE surgery		3 mps		6 mps		9 mps		12 mps	
	Median	IQR (Q1-Q3)	Median	IQR (Q1 – Q3)	Median	IQR (Q1 – Q3)	Median	IQR (Q1 - Q3)	Median	IQR (Q1 – Q3)
MPT (s)	12.8	4.9 (9.5 – 14.5)	11.7	4.6 (8.9 – 13.5)	12.1	6.1 (6.2 – 12.3)	12.6	1.7 (11.1 – 12.8)	12.7	10.2 (7.7 – 17.9)
*fo* (Hz)	169. 1	38.9 (146.1 – 184.9)	155.3	43.8 (138.7 – 182.5)	173.1	43.3 (148.7 – 192)	161.6	71.9 (125.3 – 197.2)	158.4	30.9 (147.2 – 178.1)
SD*fo*	2.3	10.9 (2 – 12.9)	2.4	1.7 (1.7 – 3.4)	4.5	10.8 (2 – 12.8)	4.4	6.2 (2.9 – 9.1)	5.2	12.6 (2.3 – 14.9)
Intensity (dB)	61.6	13 (54.1 – 67.1)	56.2	12.8 (53.8 – 66.6)	60.1	5.4 (57.1 – 62.5)	64	6.9 (59.1 – 66)	62.3	9.8 (57.7 – 67.5)
Jitter: local (%)	.37	.22 (.32 – .54)	.45	.30 (.33 – .63)	.47	.21 (.42 – .63)	.47	.36 (.40 – .76)	.43	.14 (.41 – .55)
Jitter: RAP (%)	.19	.13 (.16 – .29)	.25	.12 (.19 – .31)	.24	.11 (.23 – .34)	.21	.26 (.18 – .44)	.23	.14 (.16 – .30)
Shimmer: local (%)	2.8	1.1 (2 – 3.1)	2.9	2.6 (2.3 – 4.9)	3	1.7 (2.3 – 4)	4.2	3.1 (2.4 – 5.5)	2.7	1.9 (2 – 3.9)
NHR	.009	.011 (.006 – .017)	.013	.004 (.011 – .015)	.015	.006 (.012 – .018)	.022	.039 (.003 – .042)	.015	.037 (.008 – .045)
HNR (dB)	21	3.5 (20.3 – 23.8)	20.2	4.5 (18.3 – 22.8)	19.5	2.7 (18.1 – 20.8)	17.8	10.4 (14.7 – 25)	20.3	8.3 (15.9 – 24.2)
CPPs (dB)	16.5	2.9 (15 – 17.9)	17.5	4.2 (13.6 – 17.8)	16.6	2.9 (15 – 17.9)	15.9	2.7 (13.6 – 16.3)	16.4	4.9 (14.7 – 19.6)
LTAS-slope (dB)	-28.3	4.2 ([-30.6] – [-26.4])	-27.8	3.1 ([-28.4] – [-25.3])	-28.4	2.3 ([-29] – [-26.7])	-27	4.7 ([-31.2] – [-26.5])	-30.8	3.3 ([-32.1] – [-28.8])
LTAS-t-t (dB)	-18.1	11.3 ([-22.5] – [-11.2])	-15.2	17.4 ([-26.4] – [-9])	-19.5	9.5 ([- 20.7] – [-11.2])	-24.4	12.2 ([-30.3] – [-18.1])	-24	9 ([-30] – [-21])
CIW (%)	89.5	13.3 (80.3 – 93.6)	81	22.2 (72.2 – 94.4)	77.8	26.5 (62.8 – 89.3)	77.9	34.9 (51.1 - 86)	83.4	22.5 (70.4 – 92.9)
GI	8	2 (7 – 9)	8	2 (6 – 8)	7	1 (7 – 8)	7	1 (6 – 7)	7	4 (4 – 8)
SR (sil/s)	5.8	1.4 (4.8 – 6.2)	5.9	1.7 (4.6 – 6.3)	5.5	1.2 (5.1 – 6.3)	5.9	0.8 (5.4 – 6.2)	5.9	1 (5.2 – 6.2)
/pa/ (sil/s)	6.4	0.8 (6.1 – 6.9)	6.1	0.5 (5.8 – 6.3)	6.2	0.9 (5.5 – 6.4)	5.6	1 (5.1 – 6.1)	5.8	0.9 (5.2 – 6.1)
/ta/ (sil/s)	6.3	1.0 (5.8 – 6.8)	5.6	1.1 (5.4 – 6.5)	5.8	1.2 (5.2 – 6.4)	5.4	1.1 (5.1 – 6.2)	5.5	1.7 (4.7 – 6.4)
/ka/ (sil/s)	6.0	1.0 (5.6 – 6.6)	5.3	1 (4.9 – 5.9)	5.5	1.2 (4.9 – 6.1)	5.1	1.4 (4.6 – 6)	4.9	1 (4.4 – 5.4)
/pata'ka/ (sil/s)	6.7	1.0 (6.4 – 7.4)	6.7	0.4 (6.5 – 6.9)	6.4	1 (6 – 7)	6.7	0.9 (6.1 – 7)	6.1	1 (5.9 – 6.9)

Caption: IQR = interquartile range; Q1 = quartile 1; Q3 = quartile 3; mps = months post-surgery; sil/s = syllables/ second

### Reliability of SI measures

Related to intra-listener reliability, the ICC showed a high correlation between CIW at listening trial 1 (CIW-T1) and trial 2 (CIW-T2) in five of six listeners (ICC = [.73 – .98]; p < .05), with an exception in one listener (ICC = .27; p = .18). The CIW-T1/CIW-T2 difference was between 0.4 and 5.4% among listeners. Similarly, inter-listener reliability of CIW estimations was high in all three pairs of listeners (ICC = [.82 – .96]; p < .05) when CIW-T1s were considered. The difference in CIW estimations between listeners 1 and 2 of every pair of listeners was between 0.7 and 3.9%.

Similarly, the CKC revealed high intra-listener reliability between GI rating 1 (GI-R1) and rating 2 (GI-R2) in the same five listeners previously mentioned (weighted kappa estimate [WKE] = .77 - 1) and lower in the other listener (WKE = 0.39). The GI-R1/ GI-R2 difference was between 0 and 1 point on the GI scale in all listeners. GI ratings' inter-listener reliability was also high for two of three pairs of listeners when GI-R1s were considered (WKE = 0.78 – 0.89) and lower in the other pair (WKE = 0.35). The difference in GI ratings between listeners 1 and 2 of every pair of listeners was between 0.3 and 1.5 points on the GI scale. According to all these results, SI measures have an acceptable reliability.

### Group follow-up of speech

In OFF- (Table 2) and ON-med (Table 3), some SMs were lower or higher during all post-surgery assessments. Nevertheless, the Friedman test found no significant differences (p > .05) in most of the SMs (Table 4). When the test indicated significant differences (p < .05), *post ho*c analysis did not confirm them (p adjusted significance ≥ .05) (Table 4S, Supplementary Material). It had no significant differences (p < .05) between OFF-med and ON-med in any SM in pre- or post-surgery assessments (Table 5). Therefore, these results do not indicate any group speech change during follow-up.

**Table 4:** Friedman test’s results per speech measure

Speech measure	OFF-med		ON-med	
	statistic	p-value	statistic	p-value
MPT	4.09	.394	2.58	.631
*f*o	2.13	.711	1.96	.744
SD*f*o	2.22	.695	5.79	.216
Intensity	6.84	.144	5.60	.231
Jitter (local)	4.18	.382	1.01	.908
Jitter (RAP)	5.24	.263	0.38	.984
Shimmer	6.04	.196	2.84	.584
NHR	1.70	.790	2.33	.676
HNR	3.82	.431	3.02	.554
CPPS	9.16	.057	1.51	.825
LTAS-slope	1.78	.777	1.60	.809
LTAS-t-t	5.33	.255	7.73	.102
GI	14.9	.004^*^	11.8	.019*
CIW	5.61	.230	4.67	.323
SR	.44	.979	1.24	.871
/pa/	8.37	.079	7.85	.097
/ta/	10.1	.038*	6.14	.189
/ka/	13.2	.010*	20.8	.0003*
/pata'ka/	.92	.921	2.73	.604

Caption: OFF-med = OFF medication state; ON-med = ON medication state; /pa/ = alternate motion rate with /pa/; /ta/ = alternate motion rate with /ta/; /ka/ = alternate motion rate with /ka/; /pata'ka/ = sequential motion rate with /pata’ka/

* significative

**Table 5:** Wilcoxon paired test results (OFF- versus ON-med) per speech measure

Speech measure	Pre		3mps		6mps		9mps		12mps	
	V	p	V	p	V	p	V	p	V	p
MPT	14.5	.3734	15	.7263	34.5	.1727	34.5	.1727	21	.9102
*fo*	12	.25	0	.039	14	.3594	30	.4065	23.5	.9527
SD*fo*	32	.3008	37	.0976	34	.2031	30	.4258	24	.9102
Intensity	10	.1548	13	.3008	22	1	11	.3627	26	.7344
Jitter: local	37	.09766	33	.25	10.5	.1727	27	.6523	19	.722
Jitter: RAP	29	.1415	31	.3594	10	.1544	28	.5703	14	.6241
Shimmer: local	38	.07422	31.5	.3135	13	.5286	17	.5703	12.5	.26
NHR	20	.8336	39	.054	27	.234	22	.6241	22	1
HNR	14	.3594	7	.07422	8.5	.2065	22	1	19	.7344
CPPS	6	.0578	8	.09766	24	.9056	21	.9102	36	.1232
LTAS-slope	21	.7263	17.5	.5933	25	.8203	15	.4061	26	.7344
LTAS-t-t	15	.4258	14	.3594	28	.5703	23	1	31.5	.3135
CIW	20	.8203	22	1	20	.8203	16	.4961	8	.1834
GI	9	.7825	5	.2809	0	.1489	4.5	.4902	7	.265
SR	11.5	.7349	28	.5703	21	.7256	11.5	.4002	14.5	1
/pa/	10	1	4.5	.2476	18	1	24	.4401	8.5	.3972
/ta/	11	1	4	.05747	1.5	.07314	13.5	1	5	.07969
/ka/	6	0.7874	18	1	5.5	.1755	14	.6232	17	.944
/pata'ka/)	1.5	0.07394	15.5	0.8655	13.5	1	8	.6716	18	1

Caption: Pre = pre-surgery; mps = months post-surgery; /pa/ = alternate motion rate with /pa/; /ta/ = alternate motion rate with /ta/; /ka/ = alternate motion rate with /ka/; /pata'ka/ = sequential motion rate with /pata'ka/

### Individual follow-up of speech

**Figure 1:**
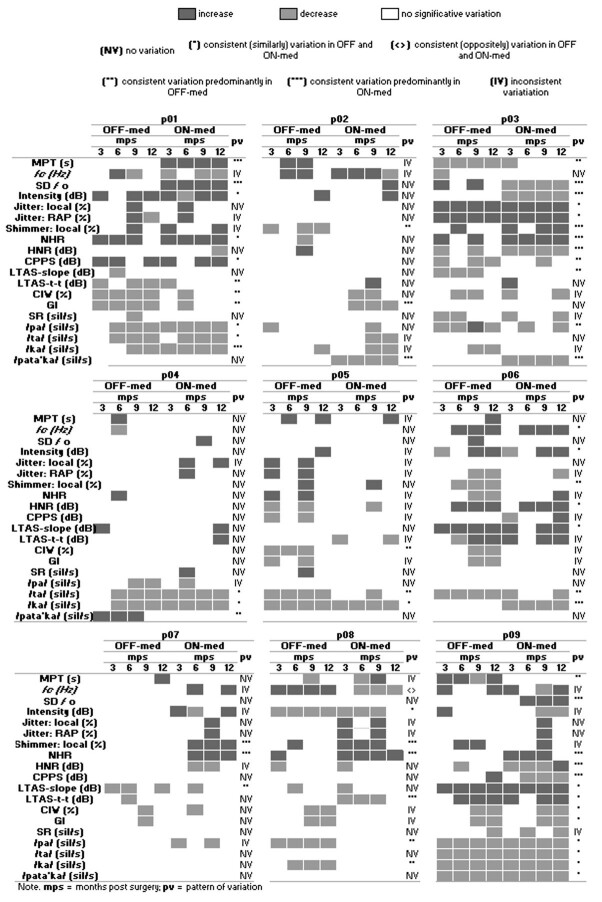
Patterns of variation of SMs per participant

A more individualized speech analysis was made because a great variability in the SMs from participant to participant was noted. These results are based on PSVIs for every participant (Tables 5S-13S, Supplementary Material). According to this analysis, the SMs showed: a) no variation (NV), b) consistent variation (CV), or c) inconsistent variation (IV) in different combinations in every participant. NV was assigned when an SM did not show any change or just varied (increased or decreased) in one of the four post-surgery speech assessments concerning pre-surgery data. CV was assigned when an SM increased or decreased during the entire or almost the entire post-surgery follow-up (3 of 4 post-surgery speech assessments). In this case, some SMs varied similarly (*) or oppositely (<>) in OFF-med and ON-med at the same time or varied exclusively in OFF-med (**) or in ON-med (***) and remained unchanged in the other medication state. IV was assigned when an SM varied after surgery without a clear pattern (Figure 1).

Some positive changes were registered during the entire or almost the entire post-surgery follow-up, in ON-, OFF-med, or both. They were an increase of MPT (p01 and p09) and voice intensity (p01), as well as a decrease of SD*f*o (p03) and Shimmer (p06) in sustained phonation. On the other hand, negative changes were related to a decrease in MPT (p03) and voice intensity (p08), as well as an increase in acoustic voice measures such as Shimmer and Jitter (p03) and NHR (p01) in sustained phonation. This classification also showed that after STN-DBS surgery, most of the SMs experienced NV or IV. In OFF-med, at 9- and 12-months post-surgery (mps), the SMs varied more frequently, and in ON-med, they made it at 6, 9, and 12 mps (Figure 1).

## DISCUSSION

This study aimed to describe STN-DBS's effects on Spanish-speaking PD patients' speech during the first year of treatment through nineteen speech measures. Although some group changes were identified after surgery, statistical analysis did not confirm them as significant in any medication state. These results agree with some previous studies in languages other than Spanish^(48-51)^ but are opposite to other similar studies' results, that in similar follow-up conditions report positive^(34-37)^ and negative^(34,39,40,63)^ post-surgery changes in SMs such as voice intensity, SD*f*o, AMR and LTAS measures (positive effects), as well as in speech intelligibility and articulation ability measures (adverse effects). Among others, three main reasons could explain the lack of group changes during follow-up: a) participants had a varied pre-surgery dysarthria level, b) a mild dysarthria level in most participants, and c) possible highly variable effects of STN-DBS on speech. These reasons are analyzed in more detail below.

### Pre-surgery varied dysarthria level

Before surgery, three levels of dysarthria were identified between subjects according to DRS-TS: low, middle, and high DRS-TS. Also, participants had SMs with normal and abnormal values at pre-surgery assessments. For example, based on available age- and sex-adapted normative data, six of nine subjects had a decreased MPT^(80)^, and four of the participants had *f*o deviations^(80)^, but Jitter (local and RAP), Shimmer, NHR, and HNR values were normal in most of them^(80)^. Similarly, CIW and GI were decreased in some patients, but oral diadochokinesis rates were normal among all subjects^(81)^. This pre-surgery heterogeneity could have contributed to the lack of an identifiable group pattern of change in speech after STN-DBS surgery.

### Mild dysarthria level predominance

Despite the three dysarthria levels classification (based on DRS-TS), most participants exhibited a relatively mild speech impairment before and after STN-DBS surgery. Similar previous research points out that when pre-surgery speech impairment is mild, the effects of STN-DBS on speech could be null^(48)^, which also can explain why participant p09, who was the participant with the worst DRS-TS before surgery, had the most remarkable change in DRS-TS alongside with a dramatic change in SI measures and oral diadochokinesis after surgery, which in turn evidence the influence of pre-surgery dysarthria level on speech outcome, as was reported by Tripoliti et al.^(63)^ who found that the worst the speech intelligibility before surgery, the worst after procedure it will be. This different result of speech outcome in participant p09 in turn could be explained by the fact that he was the only participant with cognitive compromise, which is suposed to impair the efectiviness of compensatory mechanism of speech impairment in Parkinson's disease^(41)^.

### Highly variable effects of STN-DBS on speech

The lack of speech change after surgery could also be attributable to the effects of STN-DBS itself. As was demonstrated through the individual speech measures analysis, the effects of STN-DBS in ON and OFF-med were highly variable, which makes it difficult to set up a shared pattern of change during follow-up. Almost three patterns of change of speech measures during follow-up were identified: non-variation, inconsistent variation, and consistent variation. Likewise, consistent variation along follow-up was related to ON- or OFF-med and was not the same in all participants. For example, MPT increased in OFF-med in p09 and in ON-med in p01 but decreased in p03 in OFF-med, and the same was observed in other SMs. The isolated changes in speech seen in some patients after surgery had been described in other studies: MPT increase^(46)^, voice intensity increase^(42,49)^, speech intelligibility decrease^(34,63)^, and voice acoustic measures increase^(43)^. STN-DBS settings during follow-up could ameliorate its effects on speech since all participants' pulse width and frequency stimulation were in low or mid-levels (Supplementary Material – Table 1S). These STN-DBS settings benefit speech measures such as *f*o, SD*f*o, intensity, and intelligibility^(82,83)^. Also, studies of the effects of STN-DBS on speech in extensive samples of Japanese speakers have found different speech phenotypes after stimulation onset^(29,40)^, which confirm the variability of STN-DBS effects on speech. 

Finally, other possible sources of lack of speech changes could be attributable to different years of PD and PD severity (UPDRS-III) among participants. However, the SDs of both variables were relatively low, and evidence about the influence of years of PD^(84)^ and general motor impairment^(85,86)^ on speech is not definitive. 

### Study limitations

The main limitation of this study is the lack of inclusion of a control group of non-STN-DBS treated PD patients to differentiate more strongly the effects of STN-DB on speech from typical PD-related speech degeneration. Therefore, results must be valued taking this into account. Similarly, participants of this study, as was not the case in some previous studies, kept the STN-DBS always in ON during post-surgery follow-up, so their speech characteristics when the STN-DBS was in OFF are unknown.

At the same time, because of our speech analysis methods, we cannot report vocal tremor measures, although these are commonly reported in similar studies. In the same way, the sample size does not allow the generalisation of results to the entire Spanish-speaking population with PD, and results are not fully comparable with most of the previous studies. In those studies, follow-up time was longer^(41,62-64)^ or diverse among their participants^(33,43,46,47,49)^. In other words, a longer follow-up, a larger sample, a control group, and the inclusion of vocal tremor and other speech measures with the STN-DBS in OFF are needed in future similar research in Spanish-speaking PD patients. Nevertheless, the present study results provide a detailed description of the first year of treatment outcome of STN-DBS surgery in Spanish-speaking PD patients by comparing the speech of the same patients before and after the procedure.

### Clinical implications

Our results suggest that during the first year of treatment with STN-DBS, the speech outcome could be highly variable among Spanish-speaking PD patients. Also, the results indicate that adverse changes in speech could manifest at a subclinical level since, at the end of follow-up, many of them were not detected in auditory perceptual evaluation. However, they were evident in objective speech analysis, highlighting the importance of periodic speech assessment after surgery, including subjective and objective assessment tools. In this way, speech therapy interventions must address speech impairments throughout a personalized treatment plan because these seem not heterogeneous in all Spanish-speaking PD patients after surgery, so intervention needs could vary over time.

## CONCLUSION

Results suggest that in Spanish-speaking PD patients, the effects of STN-DBS on speech are variable since a common pattern of change during the first year of treatment was not identified. However, different patterns of change in the studied speech measures were detected from participant to participant. Pre-surgery dysarthria level, as previously described, could contribute to these results as well as medication state after surgery. 

## Supplementary Material

Supplementary material accompanies this paper.

Section 1 Reading aloud task material.

Section 2 Supplementary tables

This material is available as part of the online article from https://doi.org/10.1590/2317-1782/20242023194en
