# Supplementary material for: Effects of subthalamic nucleus deep brain stimulation on the speech of Spanish-speaking Parkinson’s disease patients during the first year of treatment
Source: Codas. 2024 Sep 2;36(5):e20230194. doi: 10.1590/2317-1782/20242023194en (PMC11404841; doi:10.1590/2317-1782/20242023194en)
Supplement: Section 1 [file codas-36-5-e20230194-Suppl.pdf]

SUPPLEMENTARY MATERIAL

Effects of Subthalamic Nucleus Deep Brain Stimulation on the Speech of Spanish-speaking Parkinson's Disease Patients during the first year of Treatment

Nicolás Castillo-Triana, Maryluz Camargo-Mendoza and Oscar Bernal-Pacheco

Section 1 – Reading aloud task material.

1.1. Spanish phonetically balanced text used in the reading-aloud task.

Original text[1]

Hay algo ahí, en el aire, que cambia el sentido de las cosas. **Ese viento suave vuela, te toca la cara, mientras cuentas las hojas de los árboles (SEGMENT 1)**. El agua corre buscando los campos. Al abrir las puertas de mi casa pienso: este país, una mañana más. **A mi edad, comienzan a faltarme las fuerzas, ya casi no soy joven, y la muerte de mi mujer en la guerra me pesa mucho (SEGMENT 2)**. Cuando el cuerpo llega a esa hora, la ciencia de los doctores no logra detener el paso del tiempo. De niño, allá en mi tierra, solía pasarme los días revolviendo de un lado a otro. Poco a poco, los coches de la ciudad fueron llamando mi atención. **Mi madre decía que tuviera cuidado, pero yo me creía muy mayor, así que no tenía ni interés ni tiempo para mi propio signo (SEGMENT 3)**. Pero sigo, es cierto, cuántas cosas buenas encontré entre su gente. Si cuento los queridos veranos de entonces, no son siete, ni nueve, ni veinte. Debe ser que soy niño de nuevo en este cuerpo triste.

**** In bold, segments used to LTAS measures calculation.**

1.2. English-translated version

There is something in the air that changes the sense of things. **That smooth wind blows and touches your face while counting the leaves of the trees**. The water travels toward the fields. When I open the doors of my house, I think about this country. It is one more morning. **At my age, I feel weak, I am not so young, and I feel hard about my wife´s death in the war**. When the body comes to this time, the doctors´ science cannot stop the passing of time. When I was a child, in my town, I got used to spending my days going from one side to another. Bit by bit, the cars in the city began to engage me. **My mom told me to be careful, but I felt like an older boy, so I did not have the interest or time to take care of myself**. But I stay in. How many good things have I found among my town´s people? If I count the lovely summers of that time, there are no seven, nine, or twenty. It must be that I am again a child in this gloomy body.

---

[1] Ortega, J., González Rodríguez, J. y Marrero, V. (2000). AHUMADA: A large speech corpus in Spanish for speaker characterization and identification. *Speech Communication, 31*(2–3), 255–264. https://doi.org/10.1016/S0167-6393(99)00081-3

Section 2 – Supplementary tables

Table 1S. Participants' STN-DBS settings during follow-up.

mps	Intensity				Frequency (Hz)	
	(V)		(mA)*			
	Mean (SD)	Range	Mean (SD)	Range	Mean (SD)	Range
3	3.3 (0.6)	[2.3 – 4.4]	2.9 (0.8)	[1.7 – 3.7]	96 (24)	[60 – 130]
6	3.2 (0.5)	[2.1 – 4.1]	2.7 (1.1)	[1.5 – 3.6]	100 (28)	[60 – 139]
9	3.6 (0.6)	[2.4 – 4.7]	3.7 (0.6)	[3.0 – 4.2]	103 (32)	[60 – 139]
12	3.7 (0.7)	[2.4 – 4.7]	3.1 (1.9)	[0.7 – 4.7]	100 (32)	[60 – 139]

Note. The pulse width was constant during follow-up (60 or 70 µs); SD = standard deviation; mps = months post-surgery; V = volts; mA = milliamperes; * Intensity in mA correspond to participants with Boston Scientific device.

Table 2S. Dysarthria rating scale (DRS) dimensions[2]

Category	Speech dimensions
Pitch	1. Pitch level
	2. Pitch breaks
	3. Monopitch
	4. Voice tremor
	5. Myoclonus
	6. Diplophonia
Loudness	7. Monoloudness
	8. Excess loudness variation
	9. Loudness decay
	10. Alternating loudness
	11. Overall loudness
Voice quality	12. Harsh voice
	13. Hoarse voice
	14. Breathy voice (continuous)
	15. Breathy voice (transient)
	16. Strained-strangled voice
	17. Voice stoppages
	18. Flutter
Resonance	19. Hypernasality
	20. Hyponasality
	21. Nasal emission
	22. Weak pressure consonants

[2] Duffy. J. R. (2005, p. 90*). Motor speech disorders: substrates, differential diagnosis, and management*. Elsevier Health Sciences.

Respiration	23. Forced inspiration-expiration
	24. Audible inspiration
	25. Inhalatory stridor
	26. Grunt at end of expiration
Prosody	27. Rate
	28. Short phrases
	29. Increased rate in segments
	30. Increased rate overall
	31. Reduced stress
	32. Variable rate
	33. Prolonged intervals
	34. Inappropriate silences
	35. Short rushes of speech
	36. Excess & equal stress
Articulation	37. Imprecise consonants
	38. Prolonged phonemes
	39. Repeated phonemes
	40. Irregular articulatory breakdowns
	41. Distorted vowels
Other	42. Slow AMRs
	43. Fast AMRs
	44. Irregular AMRs
	45. Simple vocal tics
	46. Palilalia
	47. Coprolalia

The dimensions in the category 'Other' were excluded from the DRS version used in this study.

Table 3S. Participants' DRS results.

Participant	Subtotal category														Total score (TS)	
	Pitch		Loudness		Voice quality		Resonance		Breathing		Prosody		Articulation			
	P	F	P	F	P	F	P	F	P	F	P	F	P	F	P	F
p01	1	0	0	0	2	1	0	0	0	0	1	0	0	1	4	2
p02	0	0	2	2	2	1	0	0	0	0	0	0	0	0	4	3
p03	1	0	1	2	4	4	0	0	0	0	0	0	0	0	6	6
p04	0	0	0	2	1	0	0	0	0	0	0	0	0	0	1	2
p05	2	0	1	1	1	2	0	0	0	0	0	0	0	0	4	3
p06	0	2	1	0	3	2	0	0	0	0	0	0	0	0	4	4
p07	0	0	1	1	1	2	0	0	1	0	1	1	0	0	4	4
p08	1	1	2	2	2	3	0	0	1	0	2	1	0	1	8	8
p09	1	3	0	1	2	1	0	0	0	0	2	3	2	8	7	16
														Mean	4.7	5.3
														Standard deviation (SD)	2.6	4.4

Note. **P** = pre-surgery assessment; **F** = final assessment.

Table 4S. Results of post hoc analysis of speech measures with significative difference in Friedman test.

Comparison	GI				/ka/				/ta/			
	**Statistic**	**p**	**p adjusted**	**p adjusted significance**	**Statistic**	**p**	**p adjusted**	**p adjusted significance**	**Statistic**	**p**	**p adjusted**	**p adjusted significance**
OFF-med												
3-6mps	6.5	.89	1	ns	13	.933	1	ns	22	.204	1	ns
3-9mps	19	.44	1	ns	19	.944	1	ns	30.5	.092	.925	ns
3-12mps	22	.188	1	ns	21	.725	1	ns	24	.108	1	ns
3mps-pre	3	.021	.21	ns	1	.021	.207	ns	3	.075	.749	ns
6-9mps	17	.198	1	ns	12.5	.752	1	ns	21.5	.235	1	ns
6-12mps	24	.1	.99	ns	22.5	.175	1	ns	24	.108	1	ns
6mps-pre	0	.012	.11	ns	4	.059	.587	ns	5	.079	.786	ns
9-12mps	7	.577	1	ns	15.5	.344	1	ns	21	.271	1	ns
9mps-pre	0	.013	.13	ns	0	.022	.225	ns	0	.014	.14	ns
12mps-pre	0	.031	.31	ns	2	.03	.298	ns	1	.021	.207	ns
ON-med												
3-6mps	12	.824	1	ns	8	.674	1	ns				
3-9mps	21	.02	.197	ns	29	.141	1	ns				
3-12mps	18	.12	1	ns	28	.022	.223	ns				
3mps-pre	2	.17	1	ns	9	.129	1	ns				
6-9mps	18	.12	1	ns	33	.042	.421	ns				
6-12mps	19	.43	1	ns	28	.022	.22	ns				
6mps-pre	8	.17	1	ns	9	.129	1	ns				
9-12mps	1.5	1	1	ns	24	.108	1	ns				
9mps-pre	0	.034	.345	ns	10	.164	1	ns				
12mps-pre	1.5	.04	.403	ns	9	.123	1	ns				

Note. **GI** = grade of speech intelligibility; **/ka/** = alternate motion rate with /ka/; **/ta/** = alternate motion rate with /ta/; **OFF-med** = OFF medication state; **ON-med** = ON medication state; **mps** = months post-surgery; **pre** = pre-surgery; **ns** = non-significative.

Table 5S. Pre-surgery variation intervals (PSVIs) of reference for participant p01.

Speech measure	OFF-med		ON-med	
	**Mean* (SD)**	**PSVI†**	**Mean* (SD)**	**PSVI†**
MPT	14.9 (6.6)	[5 – 24.7]	14.6 (2.6)	[10.6 – 18.6]
fo	178 (19.6)	[148.7 – 207.4]	179.3 (6.7)	[148.7 – 207.4]
SD*fo*	19.9 (20.9)	[(-11.4) – 51.2]	2.1 (.5)	[1.3 – 2.9]
Intensity	54.5 (.95)	[53 – 55.9]	54.1 (.8)	[53 – 55.3]
Jitter: local	0.54 (.19)	[0.26 – .82]	.4 (.04)	[0.31 – .43]
Jitter: RAP	0.31 (.11)	[0.14 – .48]	.22 (.02)	[0.18 – .25]
Shimmer: local	3.03 (1.06)	[1.44 – 4.62]	2.42 (.51)	[1.66 – 3.18]
NHR	.008 (.007)	[(-.002) – .019]	.008 (.002)	[.004 – .011]
HNR	20.5 (1.9)	[17.6 – 23.4]	23.1 (2.6)	[19.2 – 27]
CPPS	15.1 (1)	[13.7 – 16.6]	18.4 (.9)	[16.9 – 19.8]
LTAS-slope	-24.4 (1)	[(-25.8) – (-23)]	-24.5 (2.3)	[(-28) – (-21)]
LTAS-t-t	-10.8 (11.2)	[(-27.5) – 6]	-2.2 (24.2)	[(-38.6) – 34.2]
CIW	97.5 (4.1)	[91.4 – 103.5**]	93.5 (7.8)	[81.8 – 105.2**]
GI	8.8 (.4)	[8.1 – 9.4**]	8.6 (.6)	[7.7 – 9.5**]
SR	5.9 (.7)	[4.9 – 6.9]	6 (1.6)	[3.5 – 8.4]
/pa/	7 (.4)	[6.3 – 7.6]	7.1(.2)	[6.8 – 7.4]
/ta/	6.7 (.4)	[6.1 – 7.3]	6.7 (.5)	[6 – 7.4]
/ka/	6 (.5)	[5.2 – 6.8]	7.1 (.5)	[6.4 – 7.8]
/pata'ka/	6.4 (.7)	[5.4 – 7.5]	6.6 (.7)	[5.5 – 7.7]

Note. **OFF-med** = OFF medication state; **ON-med** = ON medication state; ***** = mean value of every speech measure from pre-surgery assessment 1 and 2; **†:** mean value ± 1.5 SD; **/pa/** = alternate motion rate with /pa/; **/ta/** = alternate motion rate with /ta/; **/ka/** = alternate motion rate with /ka/; **/pataˈka/** = sequential motion rate with /pataˈka/; **(**)** = theoretical value.

Table 6S. Pre-surgery variation intervals (PSVIs) of reference for participant p02.

Speech measure	OFF-med		ON-med	
	**Mean* (SD)**	**PSVI†**	**Mean* (SD)**	**PSVI†**
MPT	12.4 (1)	[10.9 – 13.9]	14.6 (1.9)	[11.7 – 17.5]
fo	187.6 (15.1)	[164.9 – 210.2]	189.9 (9.8)	[164.9 – 210.2]
SD*fo*	11.6 (16.8)	[(-13.6) – 36.7]	8.3 (10)	[(-6.6) – 23.3]
Intensity	54.3 (4.7)	[47.2 – 61.4]	57.5 (4.5)	[50.7 – 64.3]
Jitter: local	.47 (.19)	[.18 – .75]	.5 (.29)	[.03 – .88]
Jitter: RAP	.26 (.12)	[.08 – .44]	.25 (.15)	[.02 – .48]
Shimmer: local	3.22 (.53)	[2.43 – 4.01]	2.44 (1.48)	[.21 – 4.66]
NHR	.006 (.002)	[.002 – .009]	.005 (.006)	[(-.004) – .014]
HNR	23.4 (1.3)	[21.4 – 25.3]	24 (1.7)	[21.5 – 26.5]
CPPS	14.4 (1.7)	[11.9 – 17]	15.8 (1.6)	[13.4 – 18.1]
LTAS-slope	-33 (2.9)	[(-37.4) – (-28.6)]	-32.3 (3.6)	[(-37.7) – (-26.9)]
LTAS-t-t	-11.5 (9.2)	[(-25.3) – 2.2]	-1 (10.5)	[(-16.8) – 14.8]
CIW	79 (39.5)	[19.8 – 138.2**]	95.5 (8.1)	[83.3 – 107.6**]
GI	7.8 (1.9)	[5 – 10.7**]	8.5 (.4)	[7.8 – 9.2**]
SR	6 (.9)	[4.7 – 7.4]	5.5 (.8)	[4.3 – 6.8]
/pa/	6.3 (.4)	[5.8 – 6.8]	6.4 (.3)	[6 – 6.8]
/ta/	7 (.4)	[6.4 – 7.6]	6.9 (.1)	[6.7 – 7.1]
/ka/	6.7 (.3)	[6.3 – 7.2]	6.7 (.2)	[6.4 – 7.1]
/pata'ka/	6.9 (.4)	[6.3 – 7.5]	7.5 (.1)	[7.4 – 7.7]

Note. **OFF-med** = OFF medication state; **ON-med** = ON medication state; ***** = mean value of every speech measure from pre-surgery assessment 1 and 2; **†:** mean value ± 1.5 SD; **/pa/** = alternate motion rate with /pa/; **/ta/** = alternate motion rate with /ta/; **/ka/** = alternate motion rate with /ka/ ; **/pataˈka/** = sequential motion rate with /pataˈka/; **(**)** = theoretical value.

Table 7S. Pre-surgery variation intervals (PSVIs) of reference for participant p03.

Speech measure	OFF-med		ON-med	
	**Mean* (SD)**	**PSVI†**	**Mean *(SD)**	**PSVI†**
MPT	9.4 (.7)	[8.3 – 10.4]	9 (2.6)	[5.2 – 12.9]
fo	193.5 (44.7)	[126.4 – 260.6]	182.9 (40)	[126.4 – 260.6]
SD*fo*	1.9 (.6)	[1 – 2.8]	30.7 (17.8)	[4 – 57.5]
Intensity	67.3 (7.8)	[55.7 – 79]	72 (2.4)	[68.4 – 75.5]
Jitter: local	0.41 (.09)	[.27 – .54]	.4 (.07)	[.26 – .48]
Jitter: RAP	0.20 (.06)	[.10 – .29]	.20 (.05)	[.11 – .28]
Shimmer: local	3.92 (2.4)	[.32 – 7.51]	4.34 (1.42)	[2.21 – 6.48]
NHR	.008 (.008)	[(-.004) – .020]	.013 (.008)	[.001 – .025]
HNR	20.6 (2.3)	[17.2 – 24.1]	22.9 (2.3)	[19.4 – 26.3]
CPPS	14.6 (.7)	[13.6 – 15.7]	16.9 (1)	[15.4 – 18.4]
LTAS-slope	-33.6 (1.9)	[(-36.5) – (-30.8)]	-30.7 (1.8)	[(-33.4) – (-28)]
LTAS-t-t	-25.3 (7.9)	[(-37.1) – (-13.5)]	-20.3 (6.4)	[(-29.8) – (-10.7)]
CIW	68.4 (18.9)	[40 – 96.7]	77.2 (25.4)	[39.2 – 115.3**]
GI	5.5 (1.9)	[2.6 – 8.4]	6.1 (2.1)	[3 – 9.2**]
SR	6.8 (1.8)	[4.1 – 9.4]	6.9 (2.4)	[3.3 – 10.5]
/pa/	7 (.3)	[6.6 – 7.5]	6.1 (2.1)	[6.4 – 7.5]
/ta/	7.1 (1.2)	[5.3 – 9]	6.2 (.1)	[6 – 6.4]
/ka/	5.7 (.2)	[5.4 – 6]	5.4 (.6)	[4.5 – 6.4]
/pataˈka/	7.7 (.3)	[7.3 – 8.1]	7.8 (.2)	[7.6 – 8]

Note. **OFF-med** = OFF medication state; **ON-med** = ON medication state; * = mean value of every speech measure from pre-surgery assessment 1 and 2; **†:** mean value ± 1.5 SD; **/pa/** = alternate motion rate with /pa/; **/ta/** = alternate motion rate with /ta/; **/ka/** = alternate motion rate with /ka/ ; **/pataˈka/** = sequential motion rate with /pataˈka/; **(**)** = theoretical value.

Table 8S. Pre-surgery variation intervals (PSVIs) of reference for participant p04.

Speech measure	OFF-med		ON-med	
	**Mean* (SD)**	**PSVI†**	**Mean* (SD)**	**PSVI†**
MPT	9.3 (3)	[4.8 – 13.8]	11.4 (1.9)	[8.7 – 14.2]
fo	222.3 (36.4)	[167.7 – 276.9]	219.7 (15.4)	[167.7 – 276.9]
SD*fo*	10.6 (20.5)	[(-20.1) – 41.3]	1.7 (.6)	[.8 – 2.7]
Intensity	57.9 (7.1)	[47.3 – 68.5]	53.5 (11.4)	[36.4 – 70.7]
Jitter: local	.38 (.18)	[.11 – .65]	.2 (.07)	[.13 – .32]
Jitter: RAP	.23 (.11)	[.06 – .40]	.12 (.05)	[.04 – .20]
Shimmer: local	3.50 (1.44)	[1.34 – 5.66]	1.6 (.68)	[.59 – 2.61]
NHR	.011 (.007)	[.001 – .021]	.014 (.013)	[(-.006) – .033]
HNR	19.9 (3.3)	[14.9 – 24.9]	22.3 (4.2)	[16 – 28.6]
CPPS	15.7 (1.5)	[13.3 – 18]	17 (3.2)	[12.1 – 21.9]
LTAS-slope	-26 (2.6)	[(-29.9) – (-22)]	-26.2 (2.8)	[(-30.3) – (-22)]
LTAS-t-t	-6.3 (19.1)	[(-35) – 22.4]	-5.8 (16.6)	[(-30.7) – 19.2]
CIW	88.1 (16.6)	[63.1 – 113**]	74(28)	[31.9 – 116**]
GI	7.8 (1.9)	[5.5 – 10.2**]	5.9 (2.6)	[2.1 – 9.8**]
SR	5.4 (.7)	[4.4 – 6.4]	5.2 (1.1)	[3.6 – 6.8]
/pa/	6.5 (.4)	[5.8 – 7.1]	6.2 (.4)	[5.6 – 6.8]
/ta/	6 (.5)	[5.3 – 6.7]	5.9 (.2)	[5.6 – 6.2]
/ka/	5.9 (.3)	[5.4 – 6.4]	5.8 (.1)	[5.6 – 6]
/pata'ka/	6.4 (.2)	[6.1 – 6.8]	6.6 (.4)	[6 – 7.3]

Note. **OFF-med** = OFF medication state; **ON-med** = ON medication state; * = mean value of every speech measure from pre-surgery assessment 1 and 2; **†:** mean value ± 1.5 SD; **/pa/** = alternate motion rate with /pa/; **/ta/** = alternate motion rate with /ta/; **/ka/** = alternate motion rate with /ka/; **/pataˈka/** = sequential motion rate with /pataˈka/; **(**)** = theoretical value.

Table 9S. Pre-surgery variation intervals (PSVIs) of reference for participant p05.

Speech measure	OFF-med		ON-med	
	**Mean* (SD)**	**PSVI†**	**Mean* (SD)**	**PSVI†**
MPT	17 (3.2)	[12.2 – 21.8]	18.1 (2.6)	[14.3 – 21.9]
fo	100 (13.8)	[79 – 120.3]	94.9 (15.8)	[79 – 120.3]
SD*fo*	8.3 (11.8)	[(-9.3) - 26]	10.2 (9.5)	[(-4) – 24.4]
Intensity	60.5 (3.4)	[55.4 – 65.6]	60.2 (9.4)	[46.1 – 74.3]
Jitter: local	.39 (.07)	[.28 – .49]	.9 (.60)	[(-.04) – 1.75]
Jitter: RAP	.19 (0.03)	[.15 – .22]	.41 (.30)	[(-.04) – .87]
Shimmer: local	2.78 (1.49)	[.55 – 5.01]	2.73 (.76)	[1.6 – 3.87]
NHR	.023 (.011)	[.006 – .039]	.049 (.039)	[(-.009) – .107]
HNR	19.7 (2)	[16.8 – 22.7]	19.6 (1.8)	[16.8 – 22.3]
CPPS	17.8 (1.4)	[15.7 – 19.9]	16.2 (2.2)	[12.9 – 19.5]
LTAS-slope	-28.6 (1.6)	[(-31) – (-26.2)]	-28.6 (2.1)	[(-31.7) – (-25.5)]
LTAS-t-t	-31. 5 (7.9)	[(-43.4) – (-19.7)]	-35.5 (5.5)	[(-43.7) – (-27.3)]
CIW	99.2 (2)	[96.1 – 102.2]	96.8 (3.6)	[91.4 – 102.2**]
GI	8.8 (.4)	[8.2 – 9.4**]	8.6 (.6)	[7.7 – 9.5**]
SR	4.1 (.8)	[3 – 5.3]	4.8 (1.3)	[2.8 – 6.8]
/pa/	5.9 (.5)	[5.2 – 6.6]	8.6 (.6)	[5 – 6.5]
/ta/	5.6 (.3)	[5.2 – 6]	6.5 (1.3)	[4.6 – 8.4]
/ka/	5.5 (.1)	[5.3 – 5.7]	5.4 (.2)	[5.1 – 5.7]
/pata'ka/	6.1 (.5)	[5.3 – 6.9]	5.9 (.3)	[5.5 – 6.4]

Note. **OFF-med** = OFF medication state; **ON-med** = ON medication state; ***** = mean value of every speech measure from pre-surgery assessment 1 and 2; **†:** mean value ± 1.5 SD; **/pa/** = alternate motion rate with /pa/; **/ta/** = alternate motion rate with /ta/; **/ka/** = alternate motion rate with /ka/ ; **/pataˈka/** = sequential motion rate with /pata'ka/; **(**)** = theoretical value.

Table 10S. Pre-surgery variation intervals (PSVIs) of reference for participant p06.

**Speech measure**	**OFF-med**		**ON-med**	
	**Mean* (SD)**	**PSVI†**	**Mean* (SD)**	**PSVI†**
MPT	10.5 (2.3)	[7 – 13.9]	12.7 (2.1)	[9.5 – 15.8]
fo	140.1 (10.9)	[123.8 – 156.5]	152.3 (3.6)	[123.8 – 156.5]
SD*f*o	2.8 (1.4)	[.7 – 5]	2.6 (1.9)	[(-.4) – 5.5]
Intensity	61.5 (2.2)	[58.1 – 64.8]	61.4 (2.5)	[57.6 – 65.2]
Jitter: local	0.74 (.21)	[.42 – 1.06]	.6 (.18)	[.38 – .91]
Jitter: RAP	0.36 (.10)	[.21 – .51]	.34 (.11)	[.17 – .51]
Shimmer: local	3.97 (.93)	[2.58 – 5.37]	3.77 (1.25)	[1.9 – 5.64]
NHR	.015 (.007)	[.004 – .025]	.019 (.010)	[.005 – .034]
HNR	18.9 (1.2)	[17.1 – 20.8]	18.8 (1.4)	[16.6 – 20.9]
CPPS	13.2 (2.6)	[9.3 – 17.2]	14.7 (.8)	[13.4 – 15.9]
LTAS-slope	-25.7 (1)	[(-27.2) – (-24.2)]	-26.3 (.8)	[(-27.6) – (-25)]
LTAS-t-t	-24.8 (2.5)	[(-28.6) – (-21.1)]	-26.7 (1.2)	[(-28.5) – (-24.8)]
CIW	92.8 (10.2)	[77.5 – 108.1]	93.5 (14.4)	[71.9 – 115**]
GI	8.7 (.6)	[7.8 – 9.6**]	8.3 (1)	[6.9 – 9.8**]
SR	6.3 (1.3)	[4.3 – 8.2]	6.3 (.5)	[5.5 – 7]
/pa/	6.8 (.4)	[6.2 – 7.4]	8.3 (1)	[6.1 – 7.4]
/ta/	6.8 (.2)	[6.5 – 7]	6.5 (.4)	[5.9 – 7.1]
/ka/	6.4 (.1)	[6.3 – 6.5]	6.3 (.6)	[5.4 – 7.2]
/pata'ka/	6 (.7)	[4.9 – 7.1]	6.5 (.9)	[5.2 – 7.9]

Note. **OFF-med** = OFF medication state; **ON-med** = ON medication state; ***** = mean value of every speech measure from pre-surgery assessment 1 and 2; **†:** mean value ± 1.5 SD; **/pa/** = alternate motion rate with /pa/; **/ta/** = alternate motion rate with /ta/; **/ka/** = alternate motion rate with /ka/; **/pataˈka/** = sequential motion rate with /pataˈka/; **(**)** = theoretical value.

Table 11S. Pre-surgery variation intervals (PSVIs) of reference for participant p07.

Speech measure	OFF-med		ON-med	
	**Mean* (SD)**	**PSVI†**	**Mean* (SD)**	**PSVI†**
MPT	5.2 (2.3)	[1.9 – 8.6]	7.8 (2)	[4.8 – 10.8]
fo	116.2 (25.2)	[78.3 – 154.1]	129.6 (11.4)	[78.3 – 154.1]
SD*f*o	4.8 (5.5)	[(-3.5) – 13]	1.9 (.5)	[1.1 – 2.8]
Intensity	62.5 (5.9)	[53.7 – 71.3]	67.2 (1.7)	[64.7 – 69.7]
Jitter: local	.61 (.32)	[.12 – 1.09]	.3 (.09)	[.16 – .43]
Jitter: RAP	.28 (.18)	[.001 – .55]	.09 (.04)	[.03 – .15]
Shimmer: local	2.99 (1.2)	[1.18 – 4.79]	1.65 (.4)	[1.05 – 2.26]
NHR	.026 (.020)	[(-.004) – .056]	.005 (.002)	[.002 – .009]
HNR	19.3 (3.4)	[14.2 – 24.4]	22.8 (2)	[19.8 – 25.8]
CPPS	17.2 (4.1)	[11 – 23.3]	21.1 (1.4)	[18.9 – 23.2]
LTAS-slope	-30.4 (1)	[(-31.9) – (-28.8)]	-27.3 (1.9)	[(-30.2) – (-24.4)]
LTAS-t-t	-23.6 (6.5)	[(-33.3) – (-13.8)]	-14.6 (8.2)	[(-27) – (-2.3)]
CIW	71.3 (23.4)	[36.2 -106.4]	84.9 (19.1)	[(-3.7**) – 31.5]
GI	7 (1.8)	[4.4 – 9.6**]	7.7 (.9)	[6.4 - 9]
SR	7.1 (1.4)	[5 – 9.1]	7.1 (2.1)	[3.9 – 10.3]
/pa/	5.8 (.8)	[4.6 – 7.1]	7.7 (.9)	[6.2 – 7.2]
/ta/	6.1 (1.1)	[4.5 – 7.7]	6.7 (.2)	[6.4 – 7]
/ka/	5.3 (.6)	[4.3 – 6.2]	6.3 (.3)	[5.9 – 6.6]
/pata'ka/	7.2 (.8)	[6 – 8.4]	7.6 (.6)	[6.7 – 8.5]

Note. **OFF-med** = OFF medication state; **ON-med** = ON medication state; ***** = mean value of every speech measure from pre-surgery assessment 1 and 2; **†:** mean value ± 1.5 SD; **/pa/** = alternate motion rate with /pa/; **/ta/** = alternate motion rate with /ta/; **/ka/** = alternate motion rate with /ka/; **/pataˈka/** = sequential motion rate with /pata'ka/; **(**)** = theoretical value.

Table 12S. Pre-surgery variation intervals (PSVIs) of reference for participant p08.

Speech measure	OFF-med		ON-med	
	**Mean* (SD)**	**PSVI†**	**Mean* (SD)**	**PSVI†**
MPT	10.8 (2.7)	[6.8 – 14.8]	7.9 (1.1)	[6.2 – 9.6]
fo	95.7 (16.6)	[70.9 – 120.6]	161.4 (22.4)	[70.9 – 120.6]
SD*fo*	24.6 (15.7)	[1 – 48.2]	22.7 (24)	[(-13.4) – 58.7]
Intensity	53.4 (2.1)	[50.3 – 56.5]	54.4 (1.1)	[52.7 – 56.1]
Jitter: local	1.47 (.85)	[0.19 – 2.75]	.4 (.22)	[.05 – .73]
Jitter: RAP	0.53 (.29)	[.09 – .96]	.21 (.12)	[.04 – .39]
Shimmer: local	7.13 (1.87)	[4.31 – 9.94]	2.09 (1.31)	[.12 – 4.06]
NHR	.058 (.044)	[(-.007) – .123]	.011 (.019)	[(-.016) – .039]
HNR	19.2 (5.9)	[10.4 – 28.1]	21.6 (4)	[15.7 – 27.6]
CPPS	12.3 (2)	[9.3 – 15.3]	14.6 (1.3)	[12.7 – 16.5]
LTAS-slope	-26.4 (1.2)	[(-28.2) – (-24.6)]	-30.3 (2.1)	[(-33.5) – (-27.1)]
LTAS-t-t	-16.1 (10.7)	[(-32.1) – (-.05)]	-19.2 (3.9)	[(-25.1) – (-13.4)]
CIW	96.2 (4.4)	[89.6 – 102.7**]	73 (32.7)	[23.9 – 122**]
GI	7.6 (1.7)	[5.1 – 10.2**]	5.9 (2.7)	[1.9 – 9.9**]
SR	5.2 (2.4)	[1.6 – 8.8]	4.6 (1.1)	[3.1 – 6.2]
/pa/	5.8 (.8)	[4.6 – 6.9]	4.5 (.8)	[3.4 – 5.7]
/ta/	5.5 (.9)	[4.1 – 6.9]	4.9 (.6)	[4.1 – 5.8]
/ka/	5.8 (.6)	[4.9 – 6.6]	4.6 (.9)	[3.3 – 5.9]
/pata'ka/	6.5 (1.3)	[4.5 – 8.5]	6.3 (1.3)	[4.3 – 8.3]

Note. **OFF-med** = OFF medication state; **ON-med** = ON medication state; ***** = mean value of every speech measure from pre-surgery assessment 1 and 2; **†:** mean value ± 1.5 SD; **/pa/** = alternate motion rate with /pa/; **/ta/** = alternate motion rate with /ta/; **/ka/** = alternate motion rate with /ka/; **/pataˈka/** = sequential motion rate with /pataˈka/; **(**)** = theoretical value.

Table 13S. Pre-surgery variation intervals (PSVIs) of reference for participant p09.

Speech measure	OFF-med		ON-med	
	**Mean* (SD)**	**PSVI†**	**Mean* (SD)**	**PSVI†**
MPT	11.6 (1.5)	[9.4 – 13.9]	14.5 (6.4)	[4.9 – 24]
fo	128.3 (9.1)	[114.6 – 142]	154.3 (3.8)	[114.6 – 142]
SD*f*o	6.5 (7.4)	[(-4.6) – 17.6]	2.3 (1)	[0.8 – 3.8]
Intensity	63.8 (2.5)	[59.9 – 67.6]	67.1 (.3)	[66.6 – 67.6]
Jitter: local	0.51 (.21)	[0.21 – .82]	.4 (.10)	[0.22 – .52]
Jitter: RAP	0.23 (.11)	[0.06 – .40]	0.18 (.05)	[0.10 – .26]
Shimmer: local	2.49 (.41)	[1.87 – 3.1]	2.96 (.53)	[2.17 – 3.74]
NHR	.026 (.027)	[(-.015) – .066]	.009 (.001)	[.008 – .011]
HNR	20.3 (1.5)	[18.1 – 22.5]	20.8 (.4)	[20.2 – 21.4]
CPPS	18.7 (.4)	[18.1 – 19.3]	17.9 (.7)	[16.9 – 19]
LTAS-slope	-28.1 (.8)	[(-29.2) – (-26.9)]	-31.7 (.7)	[(-32.7) – (-30.7)]
LTAS-t-t	-11.1 (1.5)	[(-13.3) – (-8.9)]	-16.9 (1.9)	[(-19.7) – (-14.1)]
CIW	31.5 (31.6)	[(-15.9**) – 78.9]	19.5 (3.9)	[13.6 – 25.3]
GI	3.7 (1.6)	[1.3 – 6.1]	5 (2.6)	[1 – 9]
SR	4.5 (.3)	[4.1 – 4.9]	6.1 (1.7)	[3.6 – 8.6]
/pa/	5.3 (1)	[3.8 – 6.8]	5 (2.6)	[6.3 – 8.7]
/ta/	7.1 (.9)	[5.8 – 8.5]	6.9 (.1)	[6.8 – 7.1]
/ka/	5.4 (.9)	[4.1 – 6.8]	5.5 (.6)	[4.6 – 6.4]
/pata'ka/	7.4 (.6)	[6.5 – 8.3]	7.8 (.6)	[6.9 – 8.7]

Note. **OFF-med** = OFF medication state; **ON-med** = ON medication state; * = mean value of every speech measure from pre-surgery assessment 1 and 2; **†:** mean value ± 1.5 SD; **/pa/** = alternate motion rate with /pa/; **/ta/** = alternate motion rate with /ta/; **/ka/** = alternate motion rate with /ka/; **/pataˈka/** = sequential motion rate with /pataˈka/; **(**)** = theoretical value.
